# Larval density can be used to predict genetic modifiers of glucagon signaling in *Drosophila melanogaster*

**DOI:** 10.1371/journal.pone.0302565

**Published:** 2024-08-28

**Authors:** Audrey Nicol, Malaika Ahmed, Chelsea Fischer, John G. Garces, Shana Magnus, Nay Maung, Nicholas Molisani, Sophia Petrov, Rebecca A. S. Palu

**Affiliations:** Department of Biological Sciences, Purdue University Fort Wayne, Fort Wayne, IN, United States of America; Karlsruhe Institute of Technology: Karlsruher Institut fur Technologie, GERMANY

## Abstract

Obesity is a growing concern. 42.3% of people in the U.S were considered obese between 2017–2018. Much is still unknown about the genetic components that contribute to weight gain. In humans, the hormone glucagon is a major contributor to the body’s energy regulation as it signals for the breakdown of lipids. Treatments targeting the glucagon pathway have helped patients with both weight loss and appetite suppression. Understanding the genetic modifiers of glucagon signaling and its downstream pathways could enable the development of a wider variety of effective therapeutics. In this study, we blocked the glucagon pathway in *Drosophila melanogaster* by reducing the expression of the fly ortholog of the glucagon receptor (*AKHR*). We then crossed our model to the *Drosophila* Genetic Reference Panel (DGRP) and looked for natural variation in fat content. We used variation in larval density to identify candidate modifier genes through a genome-wide association study. We then tested these modifier genes by increasing or decreasing their expression in the *AKHR* model. We screened these candidates initially with the same density assay used in the original study to narrow down to four candidate genes that substantially impacted the density of the larvae: *THADA*, *AmyD*, *GluRIIC*, and *CG9826*. We further characterized these candidates using biochemical assays to analyze stored metabolites such as triglycerides, glucose, glycogen, and protein under control, high sugar, and high fat conditions to see if the larvae are resistant to environmental changes. Our results indicate consistency between the results of the density assay and direct measurement of metabolite levels. In particular, *THADA* and *AmyD* are highlighted as interesting genes for additional study. We hope to improve our understanding of the glucagon signaling pathway, obesity, and lipid metabolism. We also aim to provide candidate genes that can be regarded as future therapeutic targets.

## Introduction

Obesity is associated with the leading causes of death worldwide: heart disease, diabetes, stroke, and even some types of cancer [1]. This problem is only getting worse, as prevalence has increased from 30.5% to 41.9% from 1999–2000 through 2017- March 2020 [1]. In the United States alone, ~ 3 in 4 adults 20 and older are considered obese or overweight [2]. Weight gain can be caused by many factors, including eating behaviors, some medicines, lack of physical activity or sleep, and genetics [2]. Most believe that obesity is on the rise due to increased consumption of energy-dense food and reduced physical activity [3]. Yet, this does not explain why some people become obese even when they are not living in obesogenic settings. Even through lifestyle changes, some find it hard to achieve sustainable weight loss. Studies that focus on adoption, twins, and families consistently find the heritability of BMI (body mass index) to be roughly 40–70% [4]. This means that body size variance is due to a contribution of roughly equal parts genetics and environment. Moreover, genome-wide association studies (GWAS) have found almost 150 genetic variants that are closely associated with body weight [5]. Many of the genes are seen to have variants associated with monogenic obesity [6]. This is likely due to the variant being at a large effect locus. In most cases, the obesity phenotype is instead a complex network of small effect loci. Risk of obesity cannot be properly determined without knowing and taking into account these small effect loci, though they are not well studied. Finding variants that are associated with weight can be beneficial in the treatment of obesity.

One process affected by genetic variation and that influences obesity is the glucagon pathway. Glucagon works with insulin to bring homeostasis to blood glucose levels. When blood glucose levels fall, glucagon is released from the alpha cells of the pancreatic islets of Langerhans [7]. Glucagon then works primarily on the liver to stimulate gluconeogenesis, ketone production, and lipolysis [8, 9]. There has been increasing interest on the treatment of obesity and type 2 diabetes using glucagon. In 1957, a study showed that glucagon was effective at appetite suppression and weight loss [10]. Later, in the late 1980s, another human study concluded that an increased amount of glucagon during insulin deficiency resulted in increased energy expenditure [11]. This notion has held true with glucagon-treated rats, which gained less weight and fat than their control counterparts, even when fed the same diet [12]. Several subsequent studies have demonstrated a reduced food intake and increase in energy expenditure with glucagon treatment [13–16]. Glucagon has been shown repeatedly to have effects on weight management, which makes it an attractive tool for weight-loss. When compared to lean individuals, malfunctioning glucagon secretion is seen more often in obese individuals with and without T2D [17]. They no longer respond appropriately to the fed state, and instead have elevated levels of glucagon at all times. A study evaluating both mice and humans reported the glucagon:insulin ratio to be more static in obese individuals as compared to lean individuals [18]. Identifying genetic modifiers of glucagon signaling could lead to a wider variety of therapeutics that assist in treating metabolic syndrome.

One system that has proven very effective as a model of glucagon signaling is the fruit fly *Drosophila melanogaster*. In *Drosophila*, the glucagon hormone receptor is conserved as the adipokinetic hormone receptor (*AKHR*). *AKHR* mutant flies have an astonishing increase in stored lipids and carbohydrates and are resistant to starvation, while their locomotion and feeding behaviors do not differ from wild-type flies [19]. In this study, we explore the impact of natural genetic variation in the *Drosophila* Genetic Reference Panel (DGRP) on obesity in a model of reduced *AKHR* expression. The DGRP consists of more than 200 fully sequenced inbred lines of flies derived from a natural population [20]. The natural genetic variation in the DGRP enables researchers to use GWA studies to find modifier genes of different pathways [20]. We have examined the impact of reduced *AKHR* expression on a subset of these strains in a preliminary analysis. Through this, we identified and characterized a preliminary set of candidate genes. By focusing on the genetic variation that increases risk of obesity, we may be able to identify candidate genes that can be developed as therapeutic targets in individualized treatment, which may ultimately help obese patients achieve sustainable weight management.

## Materials and methods

### Fly stocks and maintenance

Flies were raised at room temperature on a diet based on the Bloomington *Drosophila* Stock Center standard medium with malt. Experimental crosses were primarily maintained on a control media containing 6% yeast, 6% dextrose, 3% sucrose, and 1% agar, with 0.6% propionic acid and 0.1% p-Hydroxy-benzoic acid methyl ester in 95% ethanol included as antifungal agents. High sugar media used in modifier characterization contains 12% dextrose, 6% yeast, 6% sucrose, and 1% agar, with 0.6% propionic acid and 1% of *p*-hydroxy-benzoic acid methyl ester in 95% ethanol. High fat media contains 6% dextrose, 6% yeast, 5% stearate, 5% palmitate, 3% sucrose, and 1% agar, with 0.6% propionic acid and 1% of *p*-hydroxy-benzoic acid methyl ester in 95% ethanol.

The *AKHRi* strain used as a model of obesity is derived from a *r4-GAL4* (BDSC 33832) strain outcrossed to *w*^*1118*^ [21] combined with *UAS-AKHR* RNAi (BDSC 51710) to generate the final stock (*w-/w-; UAS-AKHR-RNAi/UAS-AKHR-RNAi; r4-GAL4/r4-GAL4*). 31 strains from the DGRP were used for the modifier screen (S1 and S2 Tables), wherein virgin females carrying the model were crossed to males of the DGRP strains on egg caps containing grape juice media (sucrose, agarose, concentrated grape juice, propionic acid, and p-Hydroxy-benzoic acid methyl ester) and yeast paste (yeast and water). Hybrid larvae carrying *AKHRi* were collected at the L1 stage and aged on control media at a density of 75–100 larvae per vial until the wandering L3 stage. For candidate modifier validation and characterization, virgin females carrying the *AKHRi* model were crossed to males carrying the RNAi strains on egg caps containing grape juice media and yeast paste. Hybrid larvae carrying *AKHRi* and the relevant modifier RNAi were collected at the L1 stage and aged on control, high sugar, or high fat media at a density of 75–100 larvae per vial until the wandering L3 stage. These larvae express RNAi against both *AKHR* and the candidate modifier gene. In the case of *THADA*, both RNAi and overexpression were tested. For expression validation, virgin females expressing the ubiquitous driver *tubulin-GAL4* (BDSC 60297) were crossed to the relevant modifier and control strains and RNA extracted at the wandering L3 stage for qPCR analysis. The following modifier and control strains are from the Bloomington *Drosophila* Stock Center: *CG43658* RNAi (32341, 28754), *THADA* RNAi (76340), *THADA* overexpression (76337), *CG9826* RNAi (51728), *GluRIIC* RNAi (25836), *Kek3* RNAi (77354), *CG45002* RNAi (67331), *CG18480* RNAi (28529), *CG8861* RNAi (29427), *UK114* RNAi (63684), *AmyD* RNAi (57561), control *attP40* (36304), and control *attP2* (36303). The *w*^*1118*^ strain was also used as control for *THADA* overexpression and RNAi.

### Density assay

The density assay was used as a proxy for the approximate fat content of larvae. Because fat is less dense, larvae that float at lower concentrations of sucrose than the average tend to be more fat. Larvae that float at higher concentrations of sucrose tend to be leaner and contain less fat. The assay was adapted from Hazegh and Reis 2016 [22]. L3 larvae found crawling on the side of the screen vials are picked using forceps and placed in a plastic petri dish containing a dampened, black filter paper and allowed to wander to clean off additional media. They were then transferred to a vial containing 10 mL of 8% sucrose solution. For the model validation assay, a 4% sucrose solution was used as an alternative. The larvae were given two minutes to equilibrate, after which floating larvae were scored as those above the 5 mL line. 250 μL of 20% sucrose was added to the vial and gently inverted 4–5 times. The larvae were once again allowed to equilibrate for two minutes, after which floating larvae were scored as those above the 5 mL line. Halfway through the incubation time, a metal rod was used to stir the top of the solution to ensure no larvae were floating due to surface tension. This was repeated until 95%-100% of the larvae were floating. The results are plotted and the FC50 (the concentration of sucrose at which 50% of the larvae are floating) was recorded.

### Phenotypic analysis and genome-wide association

For each DGRP line, FC50 was measured from 1–4 replicates of 30 wandering L3 larvae each. The P-values for association of genetic background and FC50 were calculated using one-way ANOVA on R software taking into account all experimental replicates for each hybrid strain. Average FC50 was used for the genome-wide association (GWA). GWA was performed as previously described [23–25]. DGRP genotypes were downloaded from the website, http://dgrp.gnets.ncsu.edu/. Non-biallelic sites were removed. Mean FC50 concentration for 81 experimental replicates representing 2430 individual DGRP/*AKHRi* larvae were regressed on each SNP. To account for cryptic relatedness [26, 27], GEMMA (v. 0.94) [28] was used to both estimate a centered genetic relatedness matrix and perform association tests using the following linear mixed model (LMM):

y=α+xβ+u+ϵ

$$u∼\mathrm{MVN}\_n(0,\lambda \tau^{\wedge }(‐1)K)$$

$$\epsilon ∼MVN\_n(0,\tau^{\wedge }(‐1)I\_n)$$

where, as described and adapted from Zhou and Stephens 2012, y is the n-vector of average glucose concentration for the n lines, α is the intercept, x is the n-vector of marker genotypes, β is the effect size of the marker. u is a n x n matrix of random effects with a multivariate normal distribution (MVN_n) that depends on λ, the ratio between the two variance components, τ^(-1), the variance of residuals errors, and where the covariance matrix is informed by K, the calculated n x n marker-based relatedness matrix. K accounts for all pairwise non-random sharing of genetic material among lines. ϵ, is a n-vector of residual errors, with a multivariate normal distribution that depends on τ^(-1) and I_n, the identity matrix. Genes were identified from SNP coordinates using the BDGP R54/dm3 genome build. A SNP was assigned to a gene if it was +/- 1 kb from a gene body.

### Metabolic assay sample preparation

Triglyceride, glucose, glycogen, and protein were all measured from the same simple fly lysate. Five L3 larvae were taken per sample for both the control and modifier group. The larvae was placed in a media-free microbial plate covered with damp black filter paper to clean off any remaining media. Five larvae were collected for each sample and ground in 100 uL 1X PBS using a dounce homogenizer. 10 microliters of the lysate was set aside on ice in a smaller microcentrifuge tube for the protein assay, while the rest was heat treated at 70°C for 10 minutes to allow for enzyme inactivation. The lysate can be stored at -20°C until assays are run.

### Triglyceride assay

The triglyceride assay measures fats in the form of triglycerides stored in the larvae. The larval lysate was inverted several times to ensure consistent mixing and 3 μl of whole lysate was placed in two 1.5 mL microcentrifuge tubes. For the control (-) sample, 37 μl of 1XPBS was added. For the triglyceride (+) group, 20 μl of Sigma Triglyceride Reagent (Sigma T2449) and 17 μl of 1XPBS was added to the second sample. The tubes were then incubated at 37°C for ~60 minutes. The tubes were then centrifuged at ~16,000 for five minutes at room temperature. 30 μl of supernatant for each control and triglyceride (+) samples is added to a 96 well plate. 100 μl of Free Glycerol Reagent (Sigma F6428) was added to all wells. The plate was covered with parafilm and left to incubate at room temperature for 5 minutes. The plate was then read at 540nm using a plate reader [29]. The candidate genes that used this assay are as follows: *THADA* RNAi, *AmyD* RNAi, *GluRIIC* RNAi.

Alternatively, the Glycerol Assay kit (Sigma MAK117) was used when the original reagents were unavailable. The larval lysate was inverted several times and 3 μl of lysate and 17 μl of 1XPBS was placed in a 1.5 microcentrifuge tube. 10 μl of glycerol standards and 10 μl of samples were added to a 96 well plate. A master mix was made as follows: 100 μl of assay buffer, 2 μl of enzyme mix, 1 μl of ATP, and 1 μl of dye reagent. 100 μl of master mix was added to each well. The plate was protected from light and incubated at room temperature for 20 minutes. The plate was read at 570nm using a spectrophotometer. The candidate genes that used this assay are as follows: *THADA* overexpression, *AmyD RNAi*, *GluRIIC RNAi*, and *CG9826 RNAi*. Results for both assays were consistent across at least 2 experimental replicates.

### Glucose/ glycogen assay

The glucose/ glycogen assay measures sugar in the form of circulating glucose as well as stored glycogen. The samples were centrifuged at ~16,000 for five minutes at room temperature. A 1:4 dilution of larval lysate and 1XPBS was made in a new tube. 15 μl of glucose and glycogen standards were added to a 96-well plate. 15 μl of each diluted sample were transferred into two wells, one for glucose (-) samples and the other for glycogen (+) samples. 15 μl of 1XPBS was added to all glucose (-) wells as well as the glucose standards. A mix of 0.15% Amyloglucosidase reagent (Sigma 101151) and 1XPBS was made. 15 μl of this solution was added to containing glycogen (+) and glycogen standards wells. The plate was covered with parafilm and incubated at 37°C for 60+ minutes. 100 μl of glucose assay reagent (Sigma G3293) was added to all wells. The parafilm was re-placed and the plate was incubated at room temperature for 15 minutes. The plate was then ready at 340nm using a spectrophotometer [29].

### Protein assay

Protein was measured as a proxy for size. 10 μl of non-heat treated larvae isolate was centrifuged at ~16,000 for five minutes at room temperature. Protein was measured using a 1:10 ratio of supernatant and 1XPBS. 10 μl of protein standards and 10 μl of diluted samples were added to a 96 well plate. 200 μl of Bradford Protein Reagent (Sigma B6916) was added to all wells. The plate was covered with parafilm and incubated at room temperature for 5 minutes. The plate was read at 595nm using a spectrophotometer [29].

### Expression validation

RNA was isolated from wandering L3 larvae (N = 5 larvae per sample) using the NEB Monarch RNA Isolation Kit with DNAse digestion (NEB T2010S). cDNA was generated using the ThermoFisher Verso cDNA Synthesis Kit with Olido-dT primers (Thermo Fisher AB1453). Expression of *THADA*, *AmyD*, *CG9826*, and *GluRIIC* was analyzed using qPCR (see S1 Table for primer sequences). Expression was normalized using *rpl19* expression as a control.

### Statistics

All statistical tests were run using R. ANOVA was used to compare the variation among the sample means to the variation within groups for both the screen data and the candidate modifier characterization. Multiple comparisons were accounted for in the candidate modifier characterization using a Tukey’s multiple comparison test.

## Results

### Reduced *AKHR* expression reduces larval density

To ensure *AKHRi* increases larval obesity, we compared it to its genetically matched control (S1 Fig). This enabled us to measure and see if there was a change in density and a concurrent change in triglyceride content between the *AKHRi* model and the control. We see that *AKHRi* larvae float at lower sucrose concentrations (FC50 = 6.6%, N = 30) than the genetically matched control (FC50 = 7.5%, N = 30) (S1A Fig). We also observed that these same *AKHRi* larvae have higher levels of triglycerides as compared to the genetically matched control (S1B Fig). This demonstrates that reducing expression of *AKHR* is a good model of obesity, resulting in more buoyant and fatter larvae than controls consistent with previously published results [30]. This also shows that the density assay will be an effective assay to monitor larval fat content.

### Larval density varies with genetic background when *AKHR* expression is reduced

**Fig 1:**
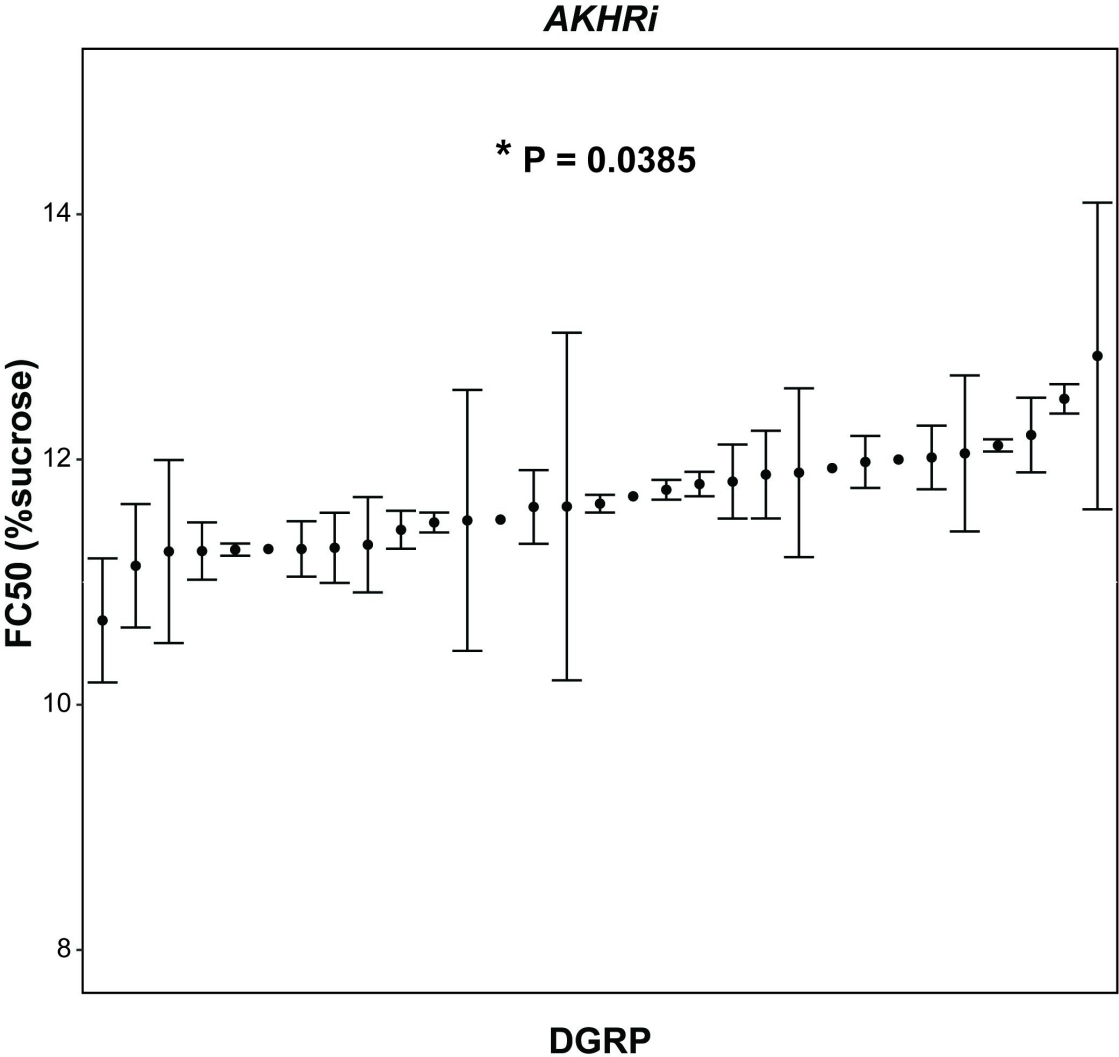
Genetic background has a significant impact on larval density in the *AKHRi* model of obesity. The concentration of sucrose at which 50% of wandering L3 larvae floated (FC50) was measured in one-four replicates of 30 larvae each for 31 strains. Average FC50 in % sucrose is indicated for each strain, with error bars indicating standard deviation (where appropriate). DGRP strains along the X-axis are ordered from lowest to highest average FC50. P-values were calculated using one-way ANOVA incorporating all individual measurements comparing DGRP strain with FC50 (P = 0.0385).

To determine if genetic background impacts obesity in the *AKHRi* model as measured by the density assay, we crossed *AKHRi* flies to 31 DGRP strains. For each assay, 30 wandering L3 larvae were exposed to increasing concentrations of sucrose until 95–100% were floating. This was repeated between one and four times for each assayed strain, with an ultimate goal of at least three replicates per strain. For each assay, the concentration of sucrose at which 50% of the larvae floated (FC50) was recorded as a quantitative readout for larval density and obesity, then averaged for each strain (Fig 1). We observed that over the 81 assays performed, genetic background had a significant impact on larval density in the *AKHRi* model (P = 0.0385).

We next used FC50 as a quantitative variable to perform a preliminary genome-wide association analysis (GWA) using the small number of assayed strains to identify potential candidate modifier genes of larval density and obesity in the *AKHRi* model (S2 and S3 Tables). GWA highlighted 213 variants associated with the phenotype at P<1*10^−4^ that lie in 84 distinct candidate genes (S4 and S5 Tables).

### Altering candidate gene expression changes larval density in the *AKHRi* model

To validate the results of the GWA, the top modifier genes for which RNAi was available (*CG43658*, *CG9826*, *GluRIIC*, *Kek3*, *CG45002*, *CG18480*, *CG8861*, *UK114)* were assayed for their impact on larval density in the *AKHRi* model. In addition, we also analyzed the impact of *THADA* and *AmyD* expression (Table 1). These candidates lie farther down the list, but have intriguing metabolic functions that warranted follow-up for their possible impacts on fat storage. *THADA* is a metabolic regulator of fat storage and heat production [31], while *AmyD* codes for an amylase which is important in starch digestion [32].

**Table 1:** Tested candidate modifier genes.

p-value	Flybase Gene ID	Gene	Function
2.39E-06	FBgn0028370	*kek3*	Plasma membrane protein
3.73E-06	FBgn0034784	*CG9826*	Transmembrane transporter
6.78E-06	FBgn0037676	*CG8861*	Neuronal cell surface glycoprotein
7.45E-06	FBgn0028518	*CG18480*	unknown
8.59E-06	FBgn0266354	*CG45002*	Proteolysis
9.30E-06	FBgn0046113	*GluRIIC*	Muscle glutamate receptor subunit
9.65E-06	FBgn0263706	*CG43658*	Rho GEF associated with ovarian cancer
1.02E-05	FBgn0086691	*UK114*	Molecular Chaperone
1.75E-05	FBgn0000078	*Amy-d*	Digestive amylase
3.45E-05	FBgn0031077	*THADA*	Regulates fat storage/heat production balance in humans

Select candidate genes for which RNAi was available were validated for their impact on larval density in the *AKHRi* model. *AmyD* and *THADA*, while not top candidates, were selected due to known metabolic functions.

**Fig 2:**
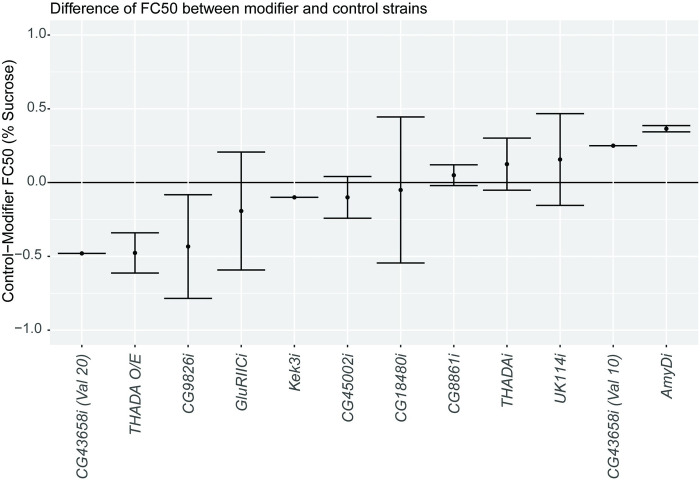
Changing expression of candidate modifier genes alters larval density in the *AKHRi* model. RNAi against candidate modifiers was expressed under the control of *r4-GAL4* in the *AKHRi* model. *THADA* was also overexpressed in the *AKHRi* model. FC50 was determined for each candidate modifier strain and compared to the FC50 for a genetically-matched control by subtracting the candidate FC50 from the control FC50. A change of 0 represents no change in FC50 or fat content (indicated by black line at y = 0). A negative change (<0) represents increased FC50 and is suggestive of reduced fat storage in the modifier strain as compared to the genetically-matched control. A positive change (>0) represents decreased FC50 and is suggestive of increased fat storage in the modifier strain as compared to the genetically-matched control. Each gene was tested in 2–4 distinct experimental replicates of 30 larvae/each.

For each candidate gene, we obtained a strain containing an RNAi construct targeted against that gene under *UAS* control. We then crossed that strain to the *AKHRi* strain, resulting in reduced expression of both *AKHR* and the candidate gene in the fat body. Two strains were available for the gene *CG43658* and both were tested. For *THADA*, overexpression was also examined by crossing a strain containing the *THADA* gene sequence under *UAS* control to the *AKHRi* strain. This resulted in overexpression of *THADA* and reduced expression of *AKHR* in the fat body simultaneously. The resulting F1 larvae were compared to genetically matched controls expressing only RNAi against *AKHR* in the fat body. FC50 for the candidate RNAi/overexpression strain was then subtracted from the FC50 for the control strain for each replicate (N = 2–4 per gene) to calculate the Change in FC50 (Fig 2 and S6 Table). A change of 0 represents no change between the control and modifier strains. A negative change (<0) represents increased FC50 and a probable reduction in fat storage in the modifier strain as compared to the genetically-matched control. A positive change (>0) represents decreased FC50 and a probable increase in fat storage in the modifier strain as compared to the genetically-matched control. Loss of *CG43658* (N = 1, Change in FC50 = 0.25: N = 1, Change in FC50 = -0.48), *Kek3* (N = 2, Change in FC50 = -0.1 ± 0.0), *CG45002* (N = 2, Change in FC50 = -0.1 ± 0.1), *CG18480* (N = 2, Change in FC50 = -0.05 ± 0.49), *CG8861* (N = 2, Change in FC50 = 0.05 ± 0.07), and *UK114* (N = 3, Change in FC50 = 0.16 ± 0.31) did not produce substantial or consistent changes in FC50 when compared to the genetically matched controls. For *CG43658*, the two RNAi strains produced opposite effects. Because the changes were not consistent, this gene was not selected for follow-up at this time. Overexpression of *THADA* (N = 3, Change in FC50 = -0.48 ± 0.14) and loss of expression of *CG9826* (N = 3, Change in FC50 = -0.43 ± 0.35) and *GluRIIC* (N = 4, Change in FC50 = -0.19 ± 0.40) all resulted in increased FC50, which is associated with reduced fat content and suppression of the obesity phenotype. For *GluRIIC*, three of the four replicates resulted in an increase in FC50, with one outlier resulting in a reduction in FC50. Loss of expression of *AmyD* (N = 2, Change in FC50 = 0.37 ± 0.02) and *THADA* (N = 2, Change in FC50 = 0.13 ± 0.18) both resulted in decreased FC50, which is associated with increased fat content and enhancement of the obesity phenotype. Importantly, loss of expression of *THADA* produced an inverse phenotype to overexpression of *THADA*, lending additional support to the impact of this modifier gene. We conclude from the initial modifier screen that we have identified a number of bona fide modifiers of larval density for the *AKHRi* model in our preliminary GWA.

### Candidate modifier have independent effects on larval density

Based on the results of the initial larval density screen of the candidate modifier genes, we selected *THADA*, *CG9826*, *GluRIIC*, and *AmyD* for additional follow-up. We confirmed change in expression for *THADA*, *AmyD*, and *GluRIIC* RNAi as well as *THADA* overexpression (S2 Fig). Expression of *CG9826* was not appreciably changed, although it appeared to trend downward. This gene was expressed at levels difficult to detect in even the wild-type samples, so while knockdown could not be confirmed analysis was continued with the other three genes.

In order to see whether the select modifier genes had an effect on their own in an otherwise wild-type background, we crossed the *CG9826*, *GluRIIC*, and *AmyD* RNAi and *THADA* overexpression modifier strains to a strain expressing only *r4-GAL4* and repeated the density assay on wandering L3 larvae. This was compared to genetically matched controls expressing only *r4-GAL4* (S3 Fig). Loss of *AmyD* alone (N = 2, Change in FC50 = 0.28 ± 0.09) or *GluRIIC* alone (N = 2, Change in FC50 = 0.83 ± 0.39) results in a decreased FC50 as compared to the control, indicating a probable increase in fat. Loss of *CG9826i* alone (N = 2, Change in FC50 = -0.33 ± 0.10) or overexpression of THADA alone ((N = 2, Change in FC50 = -0.33 ± 0.32) results in increased FC50 and a probable decrease in fat storage as compared to the genetically matched control. With the exception of *GluRIIC*, all of these effects are similar to the effect observed upon loss of these genes in the *AKHRi* model. We conclude from these results that *THADA*, *CG9826*, and *AmyD* all have independent effects on larval density and fat content, while the impact of *GluRIIC* may be due to a specific interaction with *AKHR*.

### Candidate modifier genes alter stored metabolites under a variety of environmental conditions in the *AKHRi* model

To further analyze the four candidate genes, a set of biochemical assays were used to quantify the amount of triglycerides, glucose, glycogen, and protein in L3 larva. This both serves to validate the results of the density assay and to give more information on the metabolic state of the larvae. Validation of the density assay results is critical, as this assay provides only a proxy for fat content rather than a direct measurement. RNAi and overexpression larvae in the *AKHRi* background were raised on control, high fat, and high sugar media to simultaneously examine the impact of dietary stress on metabolic homeostasis. These were compared to a genetically matched control on each diet expressing the *AKHRi* model alone.

**Fig 3:**
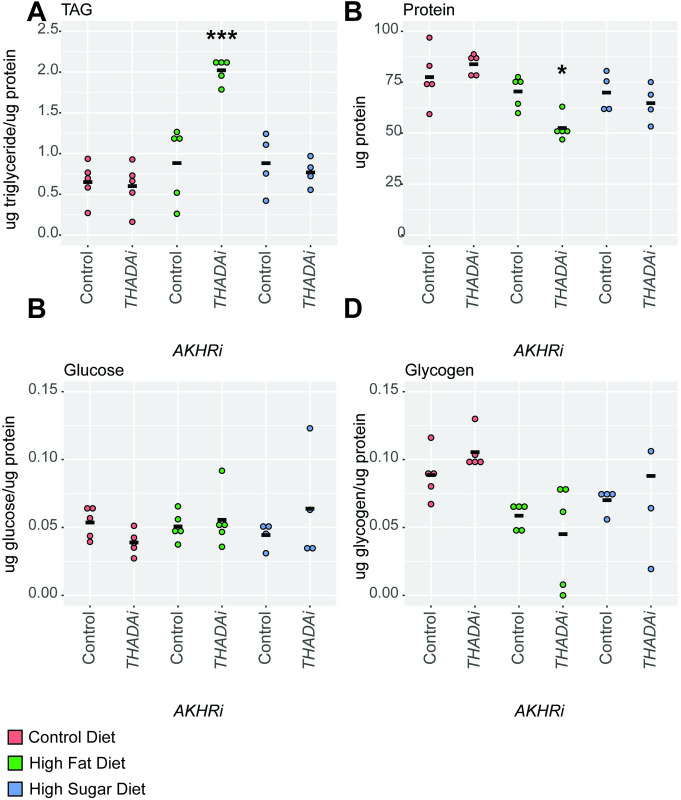
Loss of *THADA* enhances obesity in the *AKHRi* model. RNAi against *THADA* was expressed in the *AKHRi* background and compared to a genetically matched *AKHRi* control. Larvae were raised on control (red), high fat (blue), and high sugar (green) media to the wandering L3 stage under density controlled conditions (~75–100 larvae per vial). Metabolites were measured from a heat-treated lysate consisting of 5 larvae per sample and quantified for N = 4–5 samples per group. Triglycerides, glucose, and glycogen are normalized to protein levels. Protein is reported as μg protein/larva. Measurements below the detectable threshold are recorded as “0.” **A.** Triglycerides were significantly increased in *THADAi* RNAi larvae on the high fat diet as compared to the genetically-matched control on the same diet. **B.** Protein levels were significantly decreased in *THADAi* RNAi larvae on the high fat diet as compared to the genetically-matched control on the same diet. **C.** Glucose levels were unchanged in *THADAi* RNAi larvae on all three diets as compared to the genetically-matched controls on the same diets. **D.** Glycogen levels were unchanged in *THADAi* RNAi larvae on all three diets as compared to the genetically-matched controls on the same diets. P-values were calculated using one-way ANOVA followed by Tukey’s multiple testing correction. * P < 0.05, *** P < 0.005.

Under control and high sugar conditions, there is overall no change in triglyceride levels between control and *THADA* RNAi strains. However, in the high fat environment, there is a significant increase in triglycerides in *THADA* RNAi larvae (P = 0.0000708) (Fig 3A). This is consistent with the results of the density assay, which predicted a small increase in fat levels. Protein levels stayed fairly consistent, though it is slightly decreased in the *THADA* RNAi strain as compared to the control in the high fat diet (P = 0.048) (Fig 3B). No significant changes were observed in glucose or glycogen under any dietary conditions (Fig 3C and 3D).

**Fig 4:**
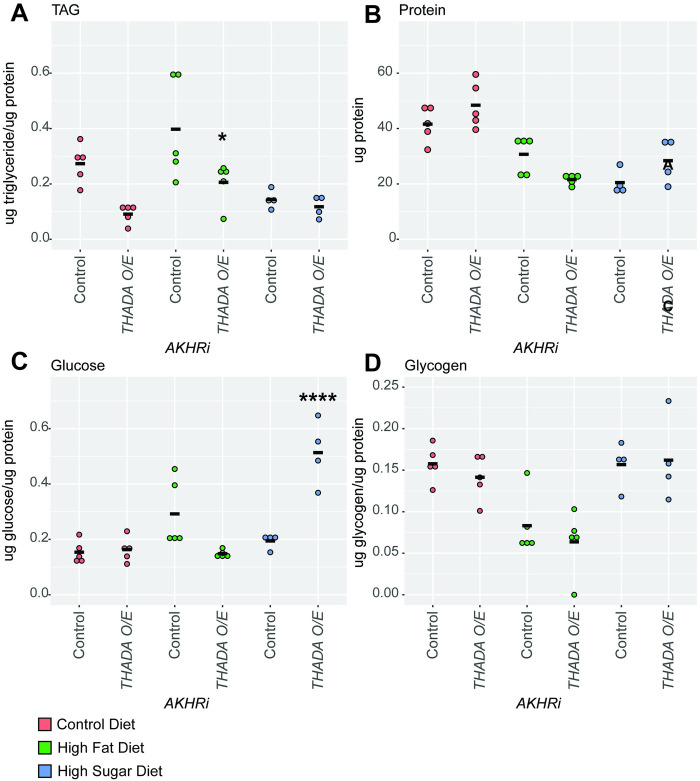
Overexpression of *THADA* suppresses obesity in the *AKHRi* model. An overexpression construct for *THADA* was expressed in the *AKHRi* background and compared to a genetically matched *AKHRi* control. Larvae were raised on control (red), high fat (blue), and high sugar (green) media to the wandering L3 stage under density controlled conditions (~75–100 larvae per vial). Metabolites were measured from a heat-treated lysate consisting of 5 larvae per sample and quantified for N = 4–5 samples per group. Triglycerides, glucose, and glycogen are normalized to protein levels. Protein is reported as μg protein/larva. Measurements below the detectable threshold are recorded as “0.” **A.** Triglycerides were increased in *THADA* overexpression larvae on both the high fat and control diets as compared to the genetically-matched control on the same diets. This change was significant on the high fat diet. **B.** Protein levels were not consistently or significantly changed in *THADA* overexpression larvae on all three diets as compared to the genetically-matched controls on the same diets. **C.** Glucose levels were significantly increased in *THADA* overexpression larvae on the high sugar diet as compared to the genetically-matched controls on the same diet. **D.** Glycogen levels were unchanged in *THADA* overexpression larvae on all three diets as compared to the genetically-matched controls on the same diets. P-values were calculated using one-way ANOVA followed by Tukey’s multiple testing correction. * P < 0.05, **** P < 0.0005.

Based on the results of the density assay, we expected *THADA* overexpression to produce opposite results as *THADA* RNAi. As predicted, upon *THADA* overexpression, triglycerides decreased as compared to the genetically matched control in both the control and high fat environment (P = 0.037 for the high fat diet). There was no change of fat content in the high sugar environment (Fig 4A). While protein levels remain fairly consistent across all diets for the control, *THADA* overexpression showed trends to decreased protein in the high fat environment, although this was not significant (Fig 4B). On a high sugar diet, *THADA* overexpression leads to a significant increase of glucose in the as compared to the genetically matched control (P = 4.8E-5) and showed no change in all other environments for glucose or glycogen (Fig 4C and 4D).

**Fig 5:**
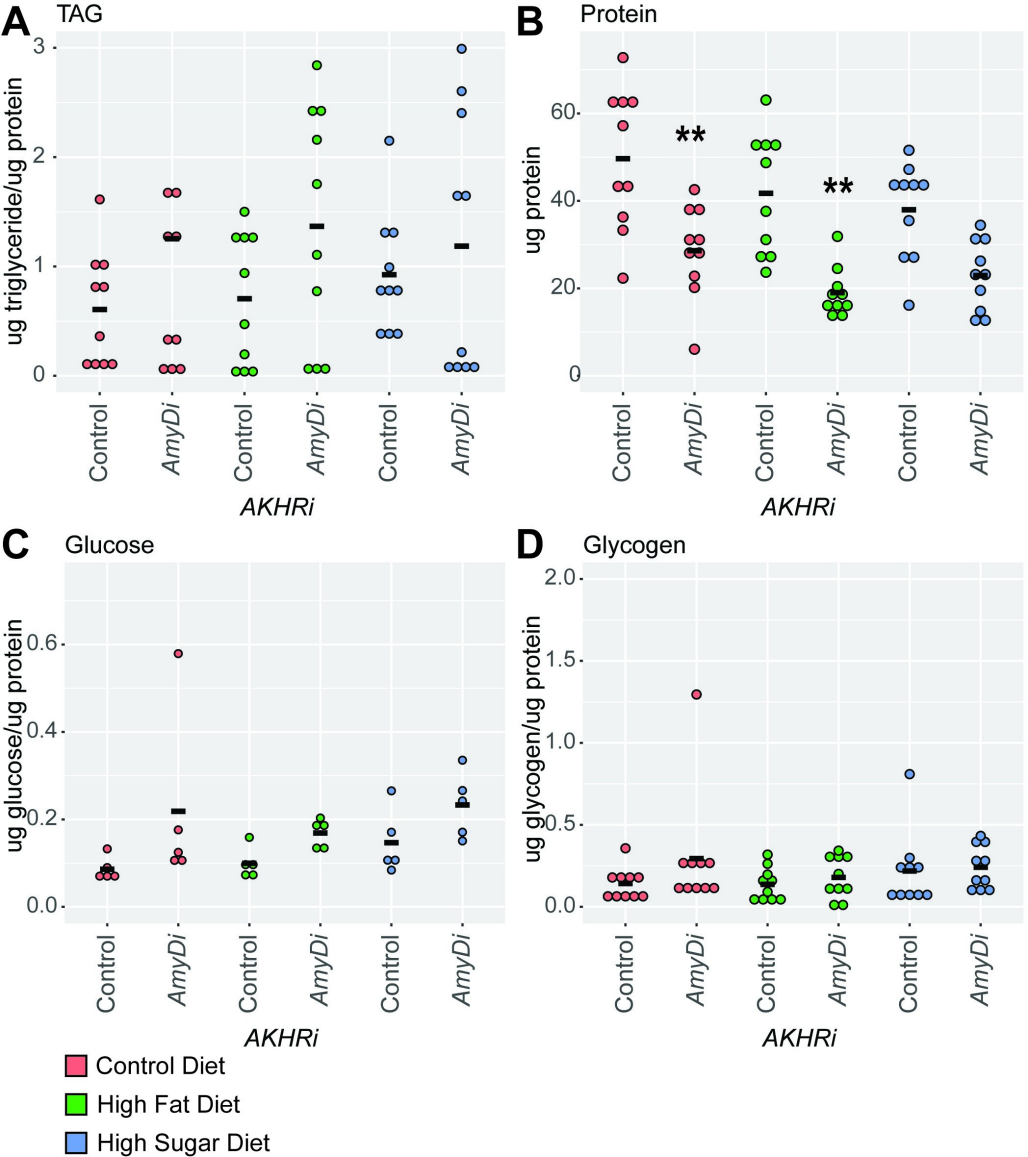
Loss of *AmyD* reduces larval size in the *AKHRi* model. RNAi against *AmyD* was expressed in the *AKHRi* background and compared to a genetically matched *AKHRi* control. Larvae were raised on control (red), high fat (blue), and high sugar (green) media to the wandering L3 stage under density controlled conditions (~75–100 larvae per vial). Metabolites were measured from a heat-treated lysate consisting of 5 larvae per sample and quantified for N = 5–10 samples per group. Triglycerides, glucose, and glycogen are normalized to protein levels. Protein is reported as μg protein/larva. Measurements below the detectable threshold are recorded as “0.” **A.** Triglycerides trended toward an increase in *AmyD* RNAi larvae on all three diets as compared to the genetically-matched controls on the same diets, although none of these changes were significant. **B.** Protein levels were decreased in *AmyD* RNAi larvae on all three diets as compared to the genetically-matched control on the same diets. This change was significant on the control and high fat diets. **C.** Glucose levels trended toward an increase in *AmyD* RNAi larvae on all three diets as compared to the genetically-matched controls on the same diets, although none of these changes were significant. **D.** Glycogen levels trended toward an increase in *AmyD* RNAi larvae on all three diets as compared to the genetically-matched controls on the same diets, although none of these changes were significant. P-values were calculated using one-way ANOVA followed by Tukey’s multiple testing correction. ** P < 0.005.

While reduced expression of *AmyD* demonstrated a trend toward increased triglycerides, glucose, and glycogen as compared to the genetically matched control under all three dietary conditions, these changes were not significant (Fig 5). This does align with the results from the density assay. However, larvae expressing *AmyD* RNAi also have significantly decreased protein when compared to the *AKHRi* control on control and high fat conditions (P = 0.0018 and 6.4E-4 respectively) (Fig 5B). As the decreased protein indicates a smaller larval size, it is possible that this drove at least in part the results of the density assay, where we may have observed *AmyDi* strains float earlier than the genetically-matched control due to their smaller size and not necessarily because of their fat content.

**Fig 6:**
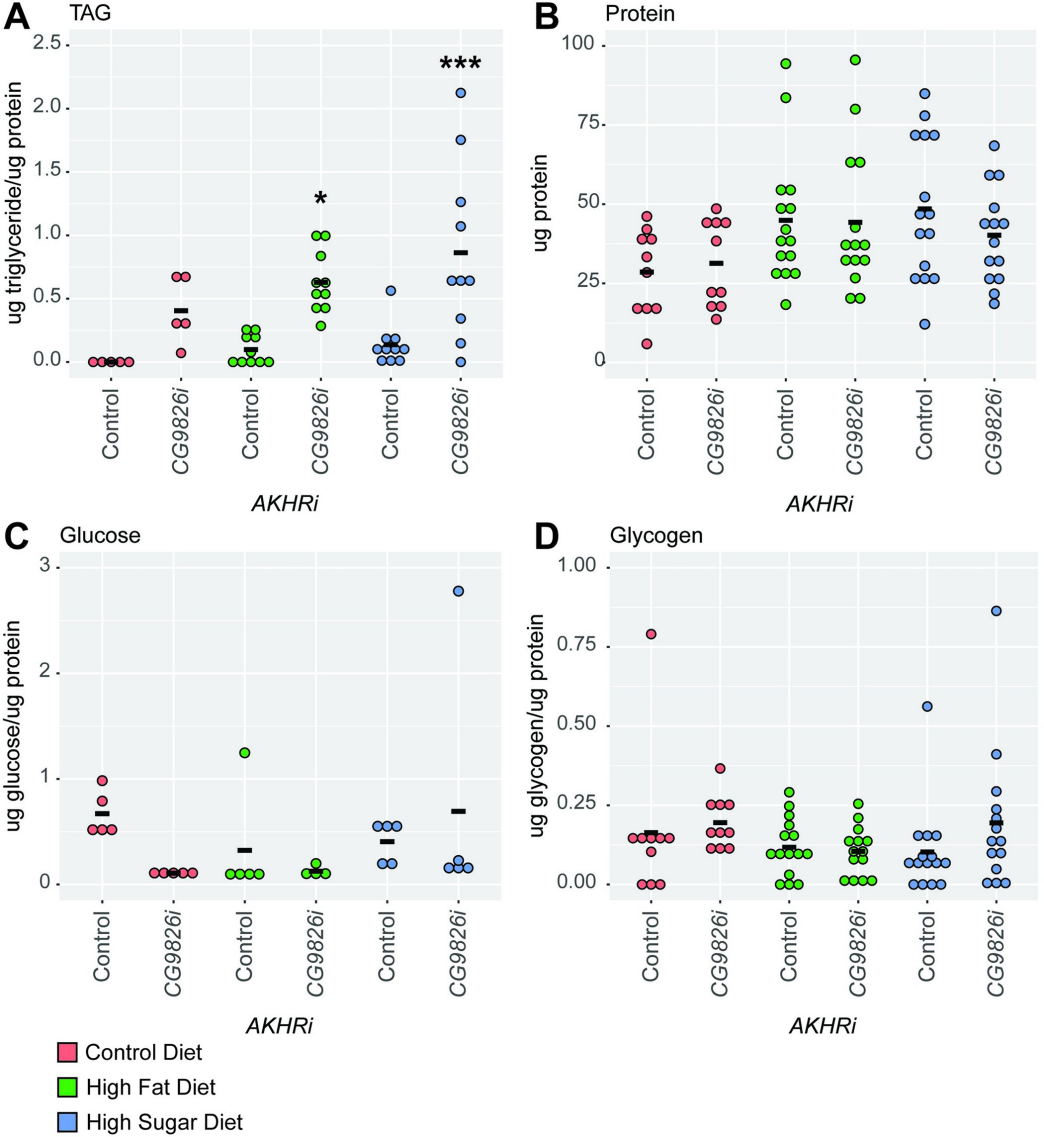
Loss of *CG9826* enhances obesity in the *AKHRi* model. RNAi against *CG9826* was expressed in the *AKHRi* background and compared to a genetically matched *AKHRi* control. Larvae were raised on control (red), high fat (blue), and high sugar (green) media to the wandering L3 stage under density controlled conditions (~75–100 larvae per vial). Metabolites were measured from a heat-treated lysate consisting of 5 larvae per sample and quantified for N = 5–15 samples per group. Triglycerides, glucose, and glycogen are normalized to protein levels. Protein is reported as μg protein/larva. Measurements below the detectable threshold are recorded as “0.” **A.** Triglycerides were significantly increased in CG9826 RNAi larvae on all three diets as compared to the genetically-matched controls on the same diets. **B.** Protein levels were unchanged in *CG9826* RNAi larvae on all three diets as compared to the genetically-matched controls on the same diets. **C.** Glucose levels were decreased in CG9826 RNAi larvae on the control diet as compared to the genetically-matched controls on the same diet, although this change was not significant. **D.** Glycogen levels were increased in *CG9826* RNAi larvae on the high sugar diet as compared to the genetically-matched controls on the same diet, although this change was not significant. P-values were calculated using one-way ANOVA followed by Tukey’s multiple testing correction. * P < 0.05, *** P < 0.0005.

Larvae predicted to have reduced *CG9826* expression had elevated triglyceride levels as compared to the genetically matched controls under both high fat and high sugar conditions (P = 0.018 and 4.5E-4 f respectively) (Fig 6A). This was unexpected, as these results are opposite what was observed in the density assay. Protein levels were unchanged (Fig 6B). There was a decrease seen in glucose for the *CG9826* RNAi line on the control media as compared to the genetically matched control, though this was not significant (Fig 6C). Glycogen levels did not substantially change for the *CG9826* RNAi strain as compared to the control strain, though an increase in glycogen can be seen in the high sugar environment (Fig 6D). The lack of consistency in these changes with the density assay may reflect inefficient knockdown of expression (S2A Fig).

**Fig 7:**
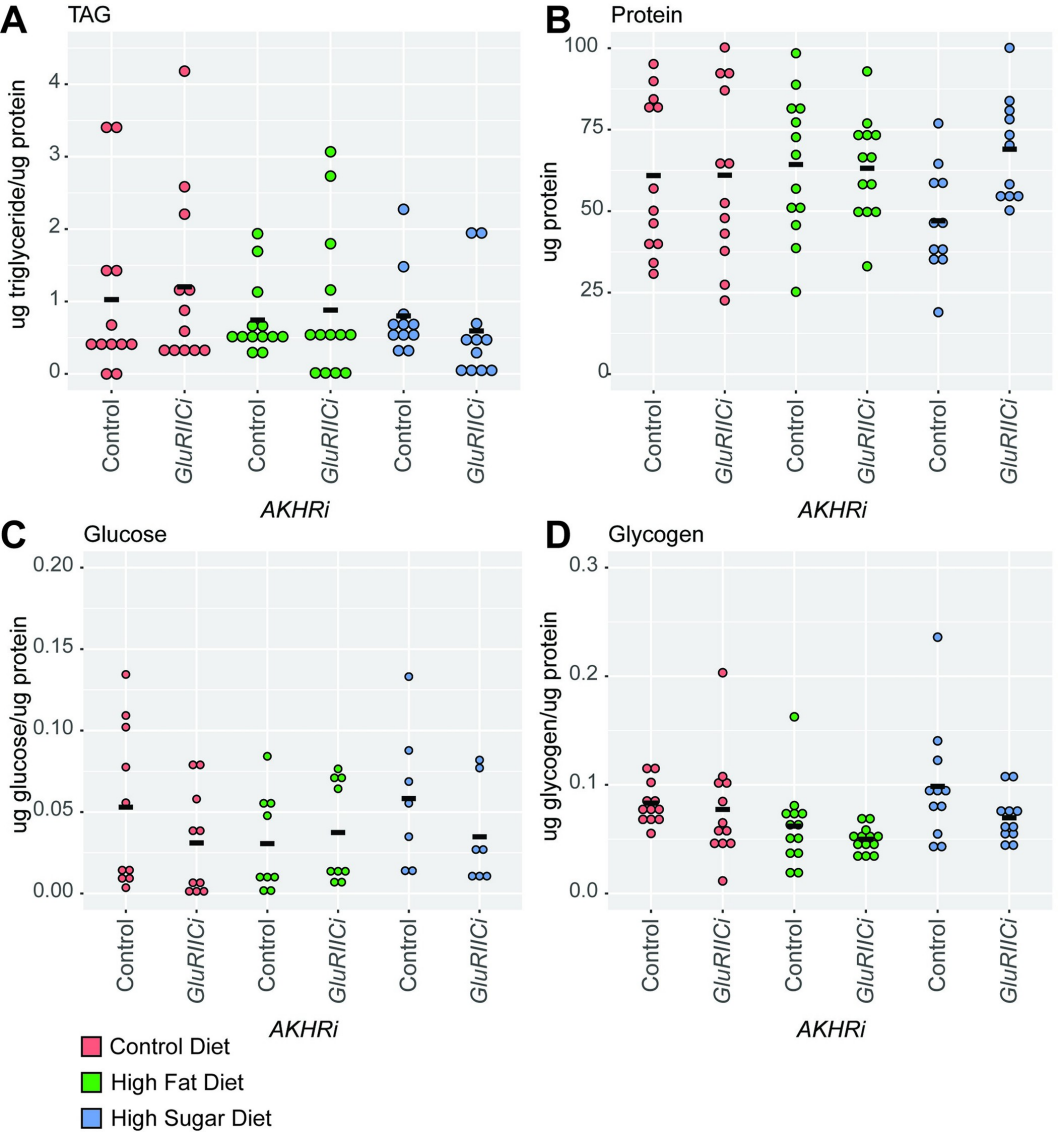
Loss of *GluRIIC* does not alter metabolic homeostasis in the *AKHRi* model. RNAi against *GluRIIC* was expressed in the *AKHRi* background and compared to a genetically matched *AKHRi* control. Larvae were raised on control (red), high fat (blue), and high sugar (green) media to the wandering L3 stage under density controlled conditions (~75–100 larvae per vial). Metabolites were measured from a heat-treated lysate consisting of 5 larvae per sample and quantified for N = 7–15 samples per group. Triglycerides, glucose, and glycogen are normalized to protein levels. Protein is reported as μg protein/larva. Measurements below the detectable threshold are recorded as “0.” **A.** Triglycerides trended toward an increase in *GluRIIC* RNAi larvae on all three diets as compared to the genetically-matched controls on the same diets, although these changes were not significant. **B.** Protein levels were unchanged in *GluRIIC* RNAi larvae on all three diets as compared to the genetically-matched controls on the same diets. **C.** Glucose levels were unchanged in *GluRIIC* RNAi larvae on all three diets as compared to the genetically-matched controls on the same diets. **D.** Glycogen levels were unchanged in *GluRIIC* RNAi larvae on all three diets as compared to the genetically-matched controls on the same diets. P-values were calculated using one-way ANOVA followed by Tukey’s multiple testing correction.

Triglyceride, glucose, glycogen, and protein levels in the *GluRIIC* RNAi larvae do not significantly differ than the genetically-matched *AKHRi* controls in any environments (Fig 7). There is a non-significant trend to increased triglycerides under all conditions when GluRIIC is reduced in expression, and this is consistent with the results of the density assay (Fig 7A). Overall, our results demonstrate that three out of four candidates originally highlighted by the density assay produce changes in metabolic state in the *AKHRi* model. These results support the use of the density assay as a reasonable proxy for changes in larval triglycerides as well as overall changes in metabolic homeostasis.

## Discussion

Obesity is a growing concern in the United States. Scientists know that both genetic and environmental factors play a role in obesity. The environmental factors are well studied but more work needs to be done to understand the genetic impact on fat storage. In this study, we utilized the glucagon pathway due to its effect on both carbohydrate and triglyceride homeostasis. Reducing expression of the glucagon hormone receptor in the flies (*AKHR*) results in increased fat storage when compared to the genetically matched control. We crossed the DGRP with our model to look for genetic variation that may increase or decrease fat storage of the larvae. While there was some variation in the FC50 within strains due to stochastic and environmental differences, the variation between different strains due to genetic background appears to be greater. We were able to use this variation to identify candidate modifier genes of larval density as a proxy for obesity. Using again the density assays, we were able to quickly screen candidate modifiers that are likely to impact fat storage. While not a perfect proxy for triglyceride levels, the density assay proved to be a fast, easy, and reproducible assay in our hands for the initial genetic modifier screen as well as for the initial candidate gene screen. This enabled us to narrow in our list of candidates to only those genes likely to have a strong impact before performing the more technical, but more precise, biochemical assays to analyze overall metabolic homeostasis.

We tested a total of ten candidate genes through the density assay compared to genetically-matched controls in the *AKHRi* obesity model background. Of these ten, four were selected for follow-up based on the consistency and size of the changes in density that were observed. For one of these, we were able to examine the impact of both increased and decreased expression (*THADA*). Our results for this gene in particular were strengthened by the expected contrast in results, where increased expression led to increased density and reduced expression led to decreased density. While we were only able to observe reduced expression for the other three genes (*CG9826*, *GluRIIC*, and *AmyD*), we went on to validate the results of the density assay for three of the four tested genes. Once again, this supports the use of this assay in the early stages of this study.

The only gene for which the density assay and metabolite levels do not agree is *CG9826*. This gene is predicted to be in the plasma membrane, involved in anion transport, and enable transmembrane transported activity [32]. Though not much is known about it, *CG9826* is predicted to be active in the plasma membrane and enable transporter activity [32]. So, it may function in the transportation or metabolism of triglycerides. Upon predicted loss of *CG9826*, larvae were overall denser than genetically matched controls during the density assay. Yet, when examining the gene further with biochemical assays, *CG9826* stored more fat than its control in all three environments. The other metabolites stayed fairly consistent with the modifier through all environments, therefore providing no explanation for this discrepancy. We did find that was not detectably changed in its expression as measured by qPCR. If expression change is inconsistent, it could account for our opposite experimental outcomes. Identifying further sources of these differences would be an interesting avenue for future exploration.

*GluRIIC* is a muscle glutamate receptor [33]. It is important for the synaptic localization of GluRIIA and GluRIIB and for synaptic transmission [34]. In humans, *GluRIIC* is closely related to *GRID 2* or glutamate ionotropic receptor delta type subunit 2 [35]. The protein encoded is ionotropic or ligand gated glutamate receptors which are the predominant excitatory neurotransmitter receptors in the brain. When glutamate binds to the receptor, a cation channel opens that allows both K+ to leave and Na+ to enter the cell. This results in an excitatory postsynaptic potential to occur which makes the postsynaptic neuron more likely to fire an action potential [36]. Under normal conditions, glutamate is the major neurotransmitter in primary perception and cognition in the brain. Excessive activation of glutamate receptors can result in excitotoxicity, a process by which neuronal dysfunction and death occurs [37]. Like *CG9826*, *GluRIIC* showed an overall reduced density in the density assay when compared to its control, which suggests an increase in larval fat. When looking at the biochemical assays, *GluRIIC* showed trends towards increased triglycerides in the control and high fat environment, though this was not significant. These trends are what we expected to see based on the density assay. Other metabolites were consistent with the control. This could be due to the location at which *GluRIIC* was reduced in expression. By reducing the expression in the muscles or the brain of the fly instead, we may be able to see more substantial and significant results. The amino acid glutamate functions in several metabolic pathways [38]. Various studies have found many diseases to have reduced skeletal muscle glutamate [38]. With this, more studies need to be done to see if there is a connection with obesity and reduced glutamate or reduced muscle glutamate receptors.

Although not top 10 candidate genes, *THADA* and *AmyD* were selected for follow-up due to their strong links with metabolic pathways. *AmyD* encodes one of three amylases, enzymes that hydrolyze dietary starches [32, 39]. The closest gene to *AmyD* in humans is amylase alpha 2B (*AMY2B)*. *AMY2B* encodes a secreted protein from the pancreas that hydrolyzes 1,4-alpha-glucoside bonds in oligosaccharides and polysaccharides. So, it is the first step in digestion of dietary starch and glycogen [40]. When used in a density assay, *AmyD* RNAi larvae showed increased fat storage. The biochemical assays did not show any significant changes, though we saw a consistent increase of triglyceride, glucose, and glycogen for all environments, as expected according to the density assay. Interestingly, the protein for *AmyD* was lower than the control for all environments. It is possible that this reduction in size of the larvae is due to a lack of starch digestion and carbohydrate homeostasis that is important during development. Physiologically, the larvae were noticeably smaller as well, which may have influenced the results of the density assay. *AmyD* RNAi strains may have floated earlier due to a combination of their small size and increased triglycerides in the *AKHRi* background. It is also possible that *AmyD* expression, which is higher in the digestive tract, is more important in that tissue than in the fat body [35]. Future characterization will involve testing the impact of *AmyD* loss in the midgut as well.

Possibly the most interesting candidate gene identified in this study is *THADA*, which encodes a regulator that synchronizes the balance between heat production and fat storage [31]. It does this by acting on the endoplasmic reticulum calcium pump [31]. In humans, the gene is also known as *THADA* through its full name is *THADA* armadillo repeat containing [35, 41]. A SNP in the human orthologue is associated with both type 2 diabetes and polycystic ovary syndrome [42, 43]. In flies, *THADA* acts on the ER calcium pump [31]. In mammals, the protein is likely to be involved in apoptosis or the death receptor pathway [44]. When looking at the density assay, larvae expressing *THADA* RNAi were reduced in density, indicating an increase in fat when compared to its control. In contrast, *THADA* overexpression resulted in increased density, indicating a decrease in fat. Direct measurement of metabolites confirmed these results. Larvae expressing *THADA* RNAi showed a great increase in triglyceride levels and a substantial decrease of protein in the high fat environment. The effects of a high fat environment on *THADAi* may highlight *THADA*’s important role in fat metabolism, and likely exacerbated the impact observed as a small effect under control conditions and in the density assay. When the regulator of fat and heat metabolism is reduced in expression, we see dysregulated fat metabolism and an increase of fat storage. *THADA* overexpression resulted in an overall decrease in triglyceride levels across the environments. Interestingly, in the high sugar environment, larvae with *THADA* overexpression had significantly more glucose than the control. This may be due to an interaction of *THADA* and *AKHR*i. With an imbalance of carbohydrate homeostasis due to *AKHRi*, *THADA* overexpression leads to overcompensating activity in the storage of glucose, especially in a high sugar environment. The impact of this should be verified in future studies.

In conclusion, we have identified various candidate modifier genes of the glucagon/ AKH pathway. We then isolated four modifiers to study further using a set of biochemical assays. Further research of these modifiers may shed light into new and therapeutics for obesity.

## Supporting information

S1 Fig Reducing *AKHR* expression in the *AKHRi* model results in reduced larval density. The *r4-GAL4* driver is used to induce expression of an RNAi construct targeting AKHR specifically in the fat body in the *AKHRi* model used in this study (black). A genetically matched control expressing only r4-GAL4 serves as the genetically-matched control strain (yellow). Larvae were aged at a density of 75–100 larvae per vial on control media to the wandering L3 stage. **A.** A larval density assay was performed simultaneously on the two strains, and the concentration of sucrose at which 50% of larvae floated was determined (FC50) based on the previously described assay (N = 30 for each genotype) [21]. Reduced larval density is associated with increased fat content, while increased larval density is associated with reduced fat content. Reduced larval density in the *AKHRi* model (FC50 = 6.6%) as compared to the genetically matched control (FC50 = 7.5%) is consistent with previous data indicating that AKHRi flies have increased fat storage [23]. This finding is consistent across 3+ experimental replicates. **B.** A triglyceride assay was performed at the same stage (N = 5 larvae per sample, 5 samples per genotype). Triglycerides were detectably elevated in the *AKHRi* larvae as compared to the genetically matched controls.
(PDF)

S2 Fig Expression of modifier genes is altered in tested strains. RNA was extracted from 5 larvae per sample (N = 3–4 samples per genotype) at the wandering L3 stage and expression analyzed by qPCR. **A.** The *tubulin-GAL4* driver is used to ubiquitously induce expression of RNAi constructs targeting *THADA*, *AmyD*, *CG9826*, and *GluRIIC* respectively in an otherwise wild-type background (blue). A genetically matched control expressing only *tubulin-GAL4* serves as the genetically-matched control strain for each construct (blue). Expression of *THADA*, *AmyD*, and *GluRIIC* were detectably reduced as compared to the genetically matched control. *CG9826* was not substantially changed. **B.** The *tubulin-GAL4* driver was also used to ubiquitously overexpress *THADA* in an otherwise wild-type background (blue) with the genetically matched control expression only *tubulin-GAL4* (red). Expression of *THADA* was detectably increased as compared to the genetically matched control.
(PDF)

S3 Fig Changing expression of candidate modifier genes alters larval density independently of genetic obesity. RNAi against *AmyD*, *CG9826*, and *GluRIIC* was expressed under the control of *r4-GAL4* in an otherwise wild-type control background. *THADA* was overexpressed under the control of *r4-GAL4*. FC50 was determined for each candidate modifier strain and compared to the FC50 for a genetically-matched control by subtracting the control FC50 from the candidate FC50. A change of 0 represents no change in FC50 or fat content (indicated by black line at y = 0). A negative change (<0) represents increased FC50 and reduced fat storage in the modifier strain as compared to the genetically-matched control. A positive change (>0) represents decreased FC50 and increased fat storage in the modifier strain as compared to the genetically-matched control. Each gene was tested in 2 distinct experimental replicates of 30 larvae/each. Loss of *AmyD* alone (N = 2, Change in FC50 = -0.28 ± 0.09) or *GluRIIC* alone (N = 2, Change in FC50 = -0.83 ± 0.39) decreases FC50 and increases fat content as compared to the genetically-matched control. Loss of *CG9826i* alone (N = 2, Change in FC50 = 0.33 ± 0.10) or overexpression of *THADA* alone (N = 2, Change in FC50 = 0.33 ± 0.32) increases FC50 and decreases fat storage as compared to the genetically matched control. With the exception of *GluRIIC*, all of these effects are similar to the effect observed upon loss of these genes in the *AKHRi* model.
(PDF)

S1 Table qPCR primers. Primer sequences used for qPCR in expression validation.
(XLSX)

S2 Table Individual FC50 (% sucrose) for each replicate in AKHRi/DGRP strains. FC50 is determined by the % sucrose at which 50% of the larvae float.
(XLSX)

S3 Table Average FC50 (% sucrose) for each AKHRi/DGRP strains. FC50 is determined by the % sucrose at which 50% of the larvae float.
(XLSX)

S4 Table Variants associated with FC50 in AKHRi larvae. All variants with a minor allele frequency of at least 0.05, p<1E-04.
(XLSX)

S5 Table Candidate modifier genes of AKHRi larval density. Variants are located within 1kb of gene region.
(XLSX)

S6 Table Candidate modifier density assay FC50 results. FC50 for paired control and modifier strains used to generate Fig 2.
(XLSX)
