# Supplementary figures and images for: Larval density can be used to predict genetic modifiers of glucagon signaling in Drosophila melanogaster
Source: PLoS One. 2024 Aug 28;19(8):e0302565. doi: 10.1371/journal.pone.0302565 (PMC11356449; doi:10.1371/journal.pone.0302565)

**A**

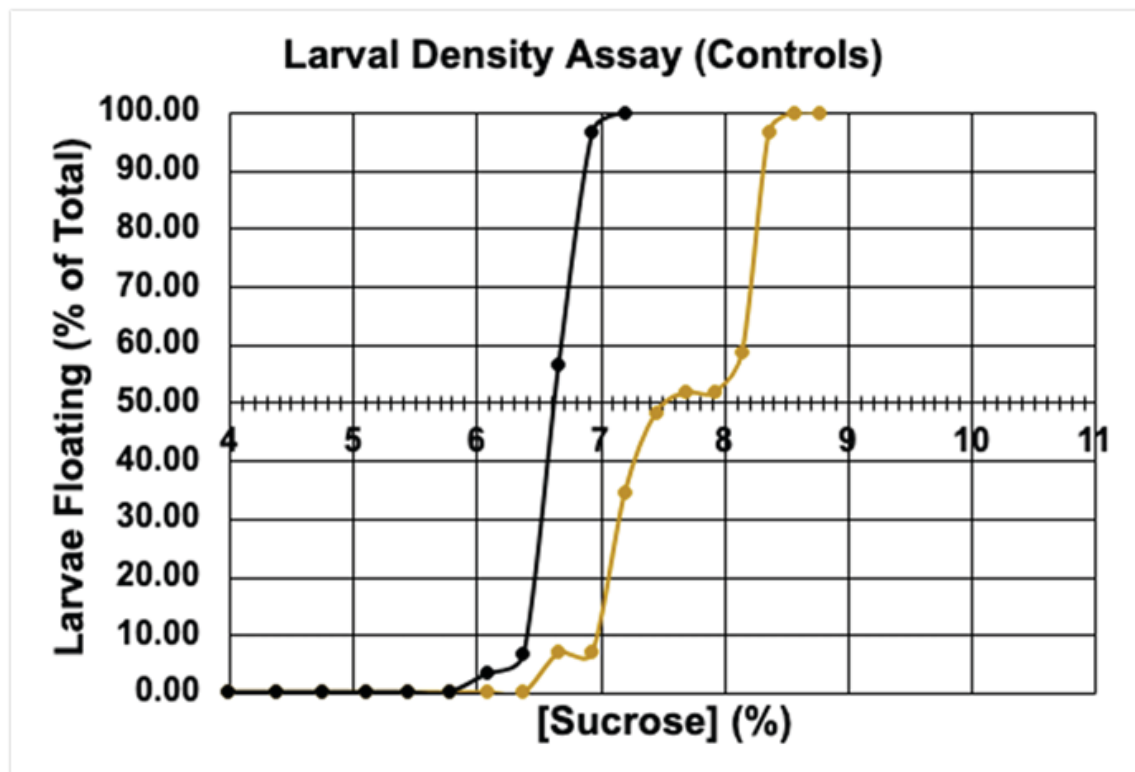

**B**

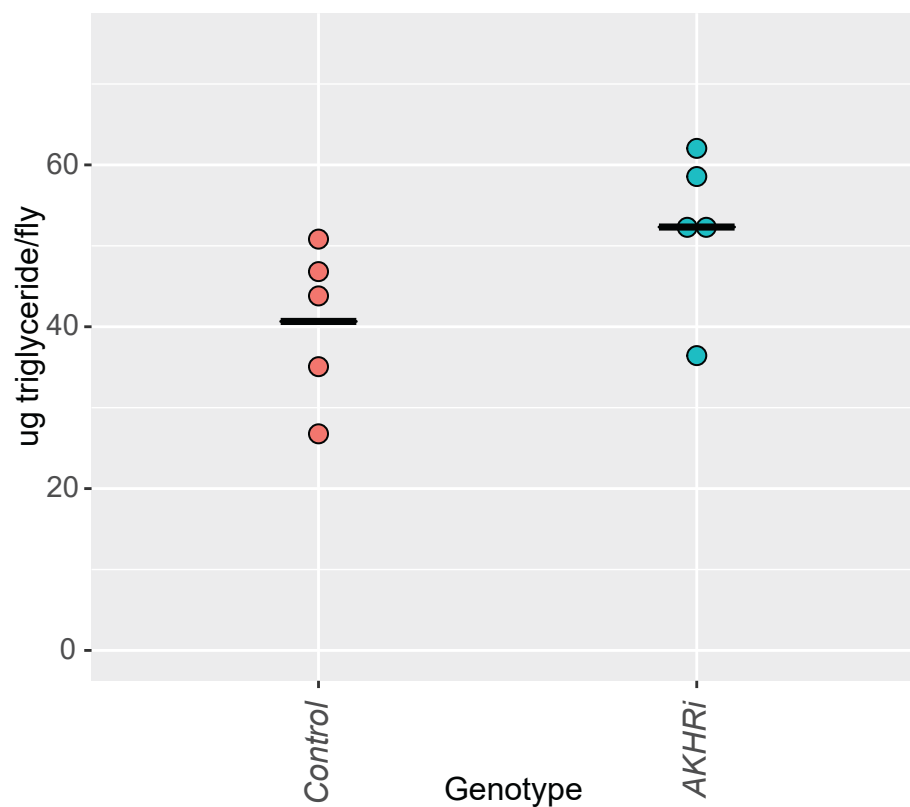

**Supplemental Figure 1**

Supplement: S1 Fig — The r4-GAL4 driver is used to induce expression of an RNAi construct targeting AKHR specifically in the fat body in the AKHRi model used in this study (black). A genetically matched control expressing only r4-GAL4 serves as the genetically-matched control strain (yellow). Larvae were aged at a density of 75–100 larvae per vial on control media to the wandering L3 stage. A. A larval density assay was performed simultaneously on the two strains, and the concentration of sucrose at which 50% of larvae floated was determined (FC50) based on the previously described assay (N = 30 for each genotype) [21]. Reduced larval density is associated with increased fat content, while increased larval density is associated with reduced fat content. Reduced larval density in the AKHRi model (FC50 = 6.6%) as compared to the genetically matched control (FC50 = 7.5%) is consistent with previous data indicating that AKHRi flies have increased fat storage [23]. This finding is consistent across 3+ experimental replicates. B. A triglyceride assay was performed at the same stage (N = 5 larvae per sample, 5 samples per genotype). Triglycerides were detectably elevated in the AKHRi larvae as compared to the genetically matched controls. (PDF) [file pone.0302565.s001.pdf]

**A**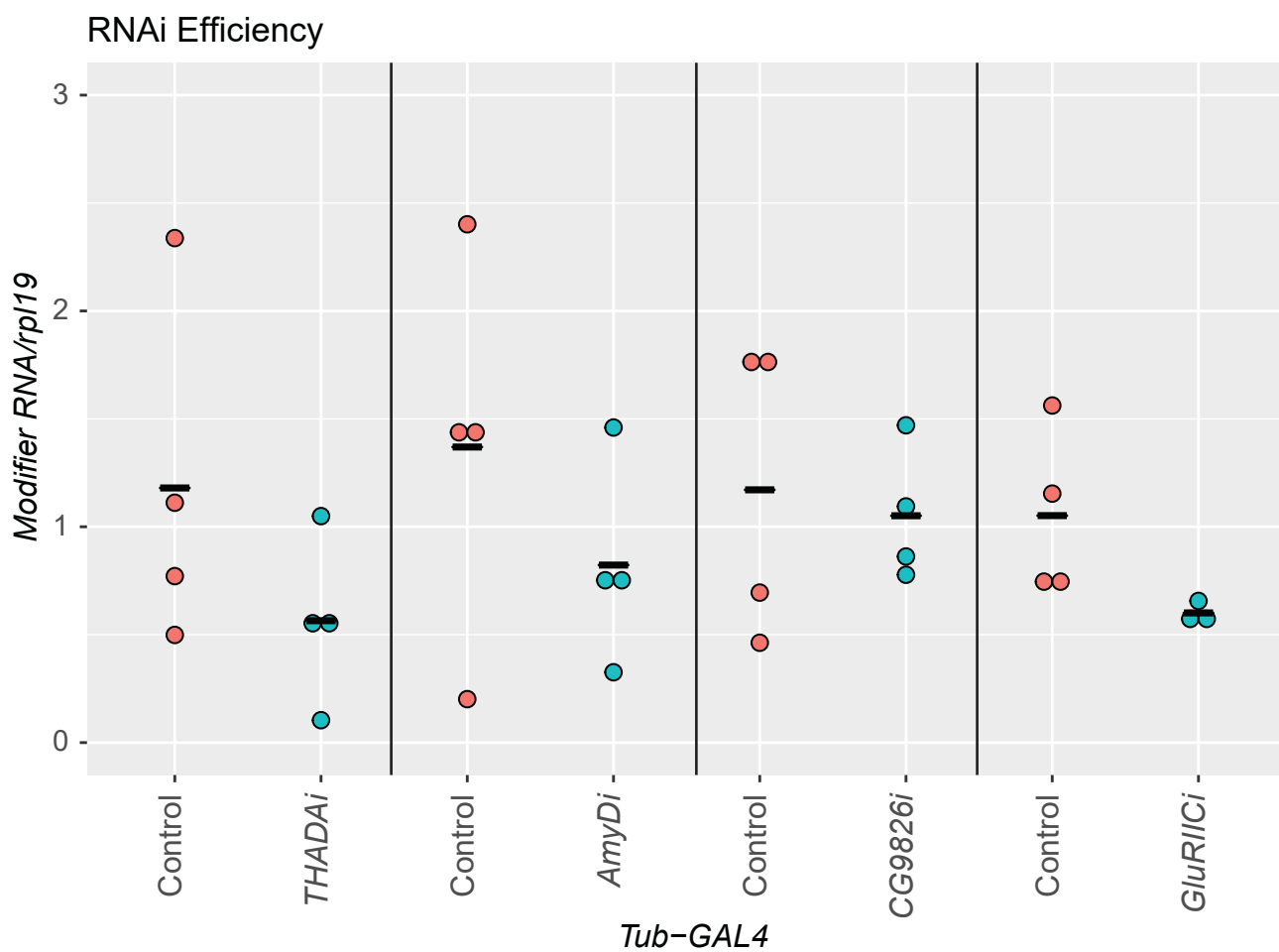**B**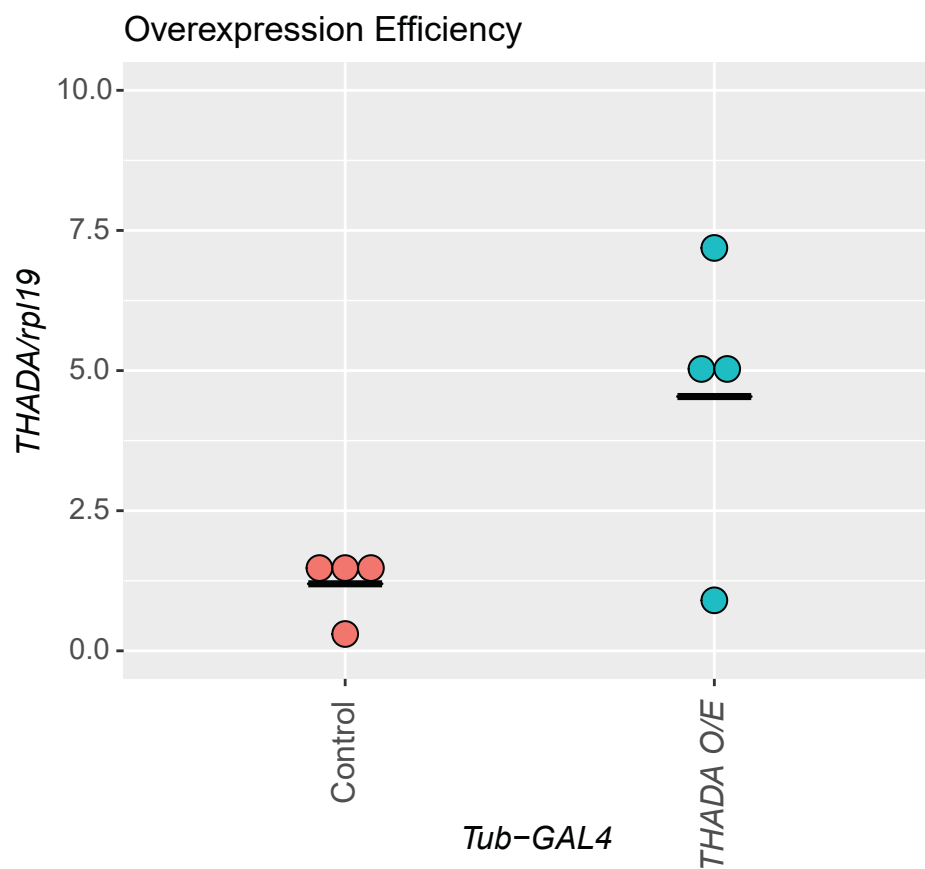**Supplemental Figure 2**

Supplement: S2 Fig — RNA was extracted from 5 larvae per sample (N = 3–4 samples per genotype) at the wandering L3 stage and expression analyzed by qPCR. A. The tubulin-GAL4 driver is used to ubiquitously induce expression of RNAi constructs targeting THADA, AmyD, CG9826, and GluRIIC respectively in an otherwise wild-type background (blue). A genetically matched control expressing only tubulin-GAL4 serves as the genetically-matched control strain for each construct (blue). Expression of THADA, AmyD, and GluRIIC were detectably reduced as compared to the genetically matched control. CG9826 was not substantially changed. B. The tubulin-GAL4 driver was also used to ubiquitously overexpress THADA in an otherwise wild-type background (blue) with the genetically matched control expression only tubulin-GAL4 (red). Expression of THADA was detectably increased as compared to the genetically matched control. (PDF) [file pone.0302565.s002.pdf]

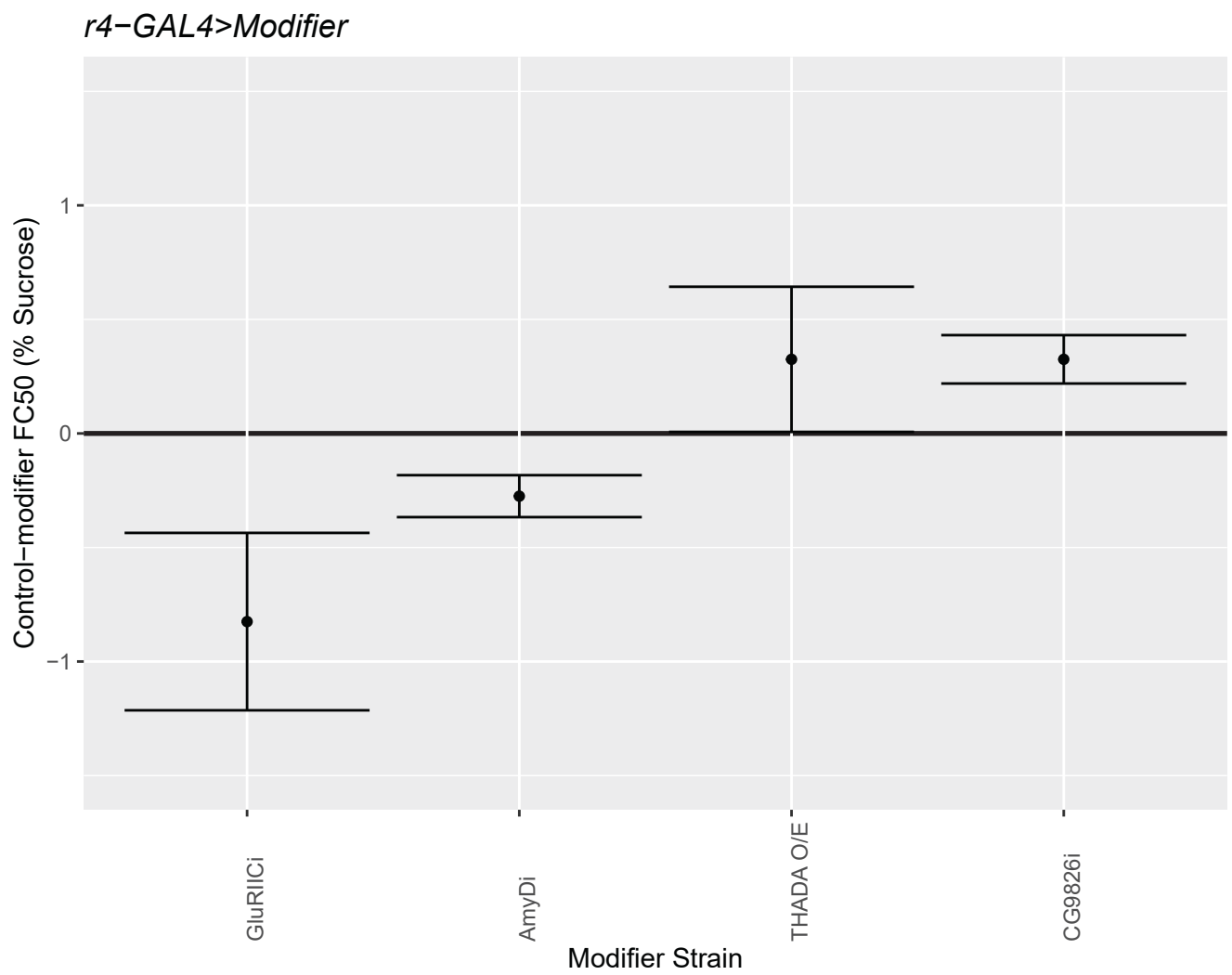

**Supplemental Figure 3**

Supplement: S3 Fig — RNAi against AmyD, CG9826, and GluRIIC was expressed under the control of r4-GAL4 in an otherwise wild-type control background. THADA was overexpressed under the control of r4-GAL4. FC50 was determined for each candidate modifier strain and compared to the FC50 for a genetically-matched control by subtracting the control FC50 from the candidate FC50. A change of 0 represents no change in FC50 or fat content (indicated by black line at y = 0). A negative change (<0) represents increased FC50 and reduced fat storage in the modifier strain as compared to the genetically-matched control. A positive change (>0) represents decreased FC50 and increased fat storage in the modifier strain as compared to the genetically-matched control. Each gene was tested in 2 distinct experimental replicates of 30 larvae/each. Loss of AmyD alone (N = 2, Change in FC50 = -0.28 ± 0.09) or GluRIIC alone (N = 2, Change in FC50 = -0.83 ± 0.39) decreases FC50 and increases fat content as compared to the genetically-matched control. Loss of CG9826i alone (N = 2, Change in FC50 = 0.33 ± 0.10) or overexpression of THADA alone (N = 2, Change in FC50 = 0.33 ± 0.32) increases FC50 and decreases fat storage as compared to the genetically matched control. With the exception of GluRIIC, all of these effects are similar to the effect observed upon loss of these genes in the AKHRi model. (PDF) [file pone.0302565.s003.pdf]
